# 

**Published:** 1894-12-15

**Authors:** 


### CONTENTS.

**Medical and Hospital Progress at Oxford** - 181
**The Anti-toxin Treatment of Diphtheria** - 182
**On Modern Progress in Ophthalmic Medicine.** By Robert Brudenell Carter, F.R.C.S. Iritis (*continued*) - 183
**The Panacea in English Medicine.** I—Theory of the Universal Medicine - 184
**Annotations.**—Religion in Hospitals—The Disappearance of the Tadpole's Tail—Experiments in Immunisation - 185
**Medical Progress and Hospital Clinics:**
On the Differential Diagnosis of Swellings in the Neck.—I. - 187
Progress in Surgery.—The Pathology of Carcinoma - 188
Tumours of the Breast - 189
Progress in Medicine.—Diseases of the Nervous System - 190
**Notes and News** - 186
**The General Medical Council** - 191
**In the Law Courts** - 192
**The Institutional Workshop:**
Hospital Construction—Falmouth Cottage Hospital - 193
Hospital Administration - 193
Hospital Meetings, &c.—Surgical Aid Society.—The Great Northern Central's Pay Wards - 195
Practical Points - 196
**Editor's Letter Box** - 196
**Scraps and Gleanings** - 196
**The Book World of Medicine and Science** - 197
**The Student of Medicine** - 197

**EXTRA SUPPLEMENT.—THE NURSING MIRROR.**

**News from the Nursing World.**—Our Christmas Competition—A New Nursing Home—Lacking Discretion—The Trained Nurses' Club—A Test Case—A Calamity Averted—Toronto Graduates—Good Nurses Appreciated—A Good Cause—Paupers and Peers—Good Works Well Done—Queen's Nurses at Wolverhampton—Friendly Co-operation—Journal-Clubs—Short Items - lxxi
**The Dietetic Management of Infancy.** II.—Change of Food - lxxiii
**Our Christmas Number** - lxxiii
**A Present for Christmas** - lxxiii
**Appointments** - lxxiii
**The Royal British Nurses' Association** - lxxiv
**Notes from India—Where to Go** - lxxiv
**The American Woman at Home.** IV.—The Dress of Some American Women - lxxv
**Presentations** - lxxv
**Everybody's Opinion** - lxxvi

